# Design and Optimization of a Self-Assembling Complex Based on Microencapsulated Calcium Alginate and Glutathione (CAG) Using Response Surface Methodology

**DOI:** 10.3390/polym13132080

**Published:** 2021-06-24

**Authors:** Ricardo I. Castro, Luis Morales-Quintana, Nancy Alvarado, Luis Guzmán, Oscar Forero-Doria, Felipe Valenzuela-Riffo, V. Felipe Laurie

**Affiliations:** 1 Multidisciplinary Agroindustry Research Laboratory, Carrera de Ingeniería en Construcción, Instituto de Ciencias Químicas Aplicadas, Universidad Autónoma de Chile, 5 Poniente 1670, Talca 3460000, Chile; 2 Multidisciplinary Agroindustry Research Laboratory, Instituto de Ciencias Biomédicas, Universidad Autónoma de Chile, 5 Poniente 1670, Talca 3460000, Chile; Luis.morales@uautonoma.cl; 3 Instituto de Ciencias Químicas Aplicadas, Facultad de Ingeniería, Universidad Autónoma de Chile, Av. El Llano Subercaseaux 2801, San Miguel, Santiago 8910060, Chile; Nancy.alvarado@uautonoma.cl; 4 Department of Clinical Biochemistry and Immunohematology, Faculty of Health Sciences, Universidad de Talca, 2 Norte 685, Talca 3460000, Chile; lguzman@utalca.cl; 5 Departamento de Ciencias Básicas, Facultad de Ciencias, Universidad Santo Tomás, Talca 3460000, Chile; oforero@santotomas.cl; 6 Programa de Doctorado en Ciencias con Mención Ingeniería Genética Vegetal, Instituto de Ciencias Biológicas, Universidad de Talca, 2 Norte 685, Talca 3460000, Chile; felipe.v.r.89@gmail.com; 7 Institute of Biological Sciences, Universidad de Talca, Campus Talca, 2 Norte 285, Talca 3460000, Chile; 8 Facultad de Ciencias Agrarias, Universidad de Talca, 2 Norte 285, Talca 3460000, Chile

**Keywords:** alginate, glutathione, microencapsulated

## Abstract

The aim of this work was to characterize and optimize the formation of molecular complexes produced by the association of calcium alginate and reduced glutathione (GSH). The influence of varying concentrations of calcium and GSH on the production of microcapsules was analyzed using response surface methodology (RSM). The microcapsules were characterized by thermogravimetric analysis (TGA-DTG) and infrared spectroscopy (FTIR) in order to assess the hydration of the complexes, their thermal stability, and the presence of GSH within the complexes. The optimum conditions proposed by RSM to reach the maximum concentration of GSH within complexes were a 15% *w/v* of GSH and 1.25% *w/v* of CaCl_2_, with which a theorical concentration of 0.043 mg GSH per mg of CAG complex was reached.

## 1. Introduction 

Oxidation reactions play an important role in food and beverage quality, ultimately being responsible for their deterioration [1]. Both vegetal and animal tissues contain antioxidant molecules that help reduce their oxidation decay, but the extent of their protection during processing or storage is limited. To overcome this problem, supplementation with exogenous antioxidants, such as glutathione, has been tested in several matrices [2,3,4]. 

Glutathione is a water-soluble, natural tripeptide composed of *N*-γ-glutamyl-cysteine-glycine that has a considerable number of free hydrophilic, amino, and carboxylic acid groups [5,6]. It is the most abundant antioxidant at the cellular level [7] and has the ability to form complexes with metals, thus limiting their catalytic activity [8,9] and moderating oxidative stress [5,7]. Glutathione has been shown to protect against phenolic oxidation, anthocyanin loss, and flavor decay in foods and beverages by reacting with quinones [2,3,4,10]. Unfortunately, the protection that GSH offers against oxidative decay is very limited, as it will quickly be lost during these types of reactions [2,10]. In fact, glutathione and other active ingredients may be sensitive to light, heat, or environmental conditions, which may reduce their bioavailability [1,11].

One alternative for the delivery of highly reactive bioactive compounds, improving their bioavailability or the protection of food surfaces, is the use of edible coatings such as alginate fibers [12,13]. Given their water solubility, they have been widely used in several applications in the pharmaceutical and food industries, including the delivery of hydrophilic nutraceuticals, β-carotene, antibiotic drugs, and other substances [14,15,16,17,18,19,20].

The properties of these types of colloidal systems have resulted in an increased scientific interest and growing market [21,22]. Some of the effects of their use in foods have to do with their safety, affinity with water, changes in sensory attributes, and storage stability of different formulations [23,24]. In addition, they have been tested as delivery systems for bioactive compounds [12,19]. 

**Figure 1:**
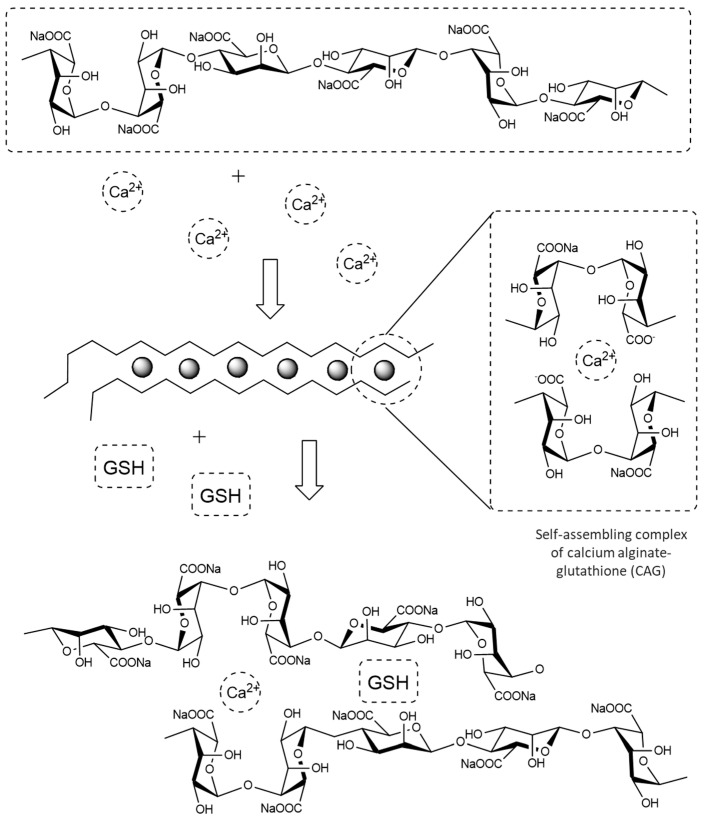
“Egg-box” model of calcium alginate upon exposure to Ca^2+^ [29] and diagram proposal of calcium alginate–glutathione complex.

Alginates are natural polymers of polysaccharides extracted from seaweeds [25,26]. They have poor mechanical properties that can be improved by mixing them with polyvalent metal ions, such as Ca^2+^ [27]. This allows the binding between alginic acid and ions to produce a spatial disposition of G and M groups in the molecular chain of alginate, in a special arrangement similar to an eggshell (see Figure 1) [28]. Encapsulating GSH may offer protection against adverse environmental conditions that could compromise the antioxidant properties of this molecule and may serve as vehicles for a potentially slower or controlled release. 

In view of all the prior data, the aim of this work was to characterize and optimize the conditions for the self-assembling formation of molecular complexes of alginate crosslinked with Ca^2+^ and GSH (CAG), using TG-DTG, FTIR, molecular simulation, and response surface methodology. 

## 2. Material and Methods 

### 2.1. Reagents 

Sodium alginate (Mw, 1.93 × 10^5^ g/mol) was obtained from Büchi Labortechnik AG, while reduced glutathione (98%) and calcium chloride (reag. Ph. Eur. ≥ 98%) were acquired from Merck. Water was produced on-site with a Milli-Q system (18 mΩ cm^−1^). 

### 2.2. Experimental Design and Preparation of the Microcapsules

A 3^2^ factorial design was tested, in which three different concentrations of GSH and CaCl_2_ were examined. The microcapsules were prepared by ionic gelation as described elsewhere [30]. A 1.5% (*w/v*) aqueous solution of sodium alginate was prepared and magnetically stirred for 12 h at ca. 25 °C. Then, different amounts of GSH were incorporated: 5%, 10%, or 15% (*w/v*). These solutions were gelated dropwise through an encapsulator B-390 BÜCHI working at a frequency of 800 Hz, 800 V electrode, 500 mbar air pressure, and nozzle of 200 µm into a CaCl_2_ solution at different concentrations: 0.75%, 1.0%, 1.25% (*w/v*) under stirring for 30 min. The microcapsules were rinsed with distilled water, sieved, and freeze dried at 4 °C. The approximate size of the nanostructures was a function of the microencapsulator used, ranging between 200 and 400 μm.

Table 1 shows the 9 combinations of reactants tested, as well as the treatment codes and replications (*n* = 3). GSH and CaCl_2_ treatments were transformed into coded units (i.e., −1, 0, and +1) to have them in a common scale and to unify their weight during the optimization analysis. The response was expressed as mg of GSH per mg of complex. 

### 2.3. FTIR Characterization of the Alginate Complexes

The Fourier transform infrared (FTIR) spectra of the CAG complexes were recorded on a Nicolet Nexus 470 spectrometer using a spectral range of 4000–500 cm^−1^. The spectra were obtained from pellets containing 100 mg of KBr and 10 mg of dry sample (i.e., each of the CAG complexes). The spectra were recorded with 32 scans using a resolution of 4 cm^−1^.

### 2.4. Thermogravimetric (TG) Characterization and Differential Thermogravimetric (DTG) Analyses of CAG Complexes 

All CAG complexes were subjected to thermogravimetric analysis with an STD 650 Thermal analyzer. For each analysis, approximately 5 mg of the mixture was placed onto a Pt crucible, and the samples were heated at a constant rate of 10 °C min^−1^ from room temperature to 550 °C, using air as a reactive gas, and with a mass flow of 50 mL min^−1^. In addition, 50 mL min^−1^ of N_2_ was used as protective gas in the electronic balance. 

### 2.5. Computational Building of the Molecular Structures and Polymeric Systems

The structure of the monomeric alginate and the protocol for computational block-building were obtained from Valdes et al., 2008 [31]. Forty alginate chains with the monomers, namely M and G ((1⟶4) linked β-D-mannopyranuronic acid and α-L-gulopyranuronic acid, respectively), were used to build GG and MM blocks that generated ten-block-long alginate chains using the LEAP module of AmberTools software [32]. The PACKMOL software [33] was used to obtain the random distribution of the 40 alginate chains, where each chain was separated by 3 to 5 Å to generate a virtual sphere of 80 Å in diameter. Finally, given that the carboxylate groups of the alginate chains are sensitive to pH changes [34,35], their protonation states were considered so as to keep around 70% of the carboxylate groups protonated. Additionally, glutathione (GSH; PubChem CID:124886) was optimized with the SCHRÖDINGER suite with the OPLS v2005 force field, specifically with the LigPrep [36] and Epik [37] tools. After ligand optimization, ten of these molecules were immersed in a water box with 0.15 mM of calcium chloride.

Along with the physical characterization of the complexes by FTIR and TG/TDG, molecular dynamic (MD) simulations of the ten GSH units in the water box containing CaCl_2_ were used to determine the possibility of obtaining GSH inside the alginate sphere. The MD simulations were studied by the NPT ensemble for 100 ns. The default relaxation protocol implemented in Desmond was used according to Castro et al. (2019) [38]. The OPLS [39] force field was applied to the systems. The resulting visualization was accomplished using the VMD software version 1.9.3 for Win32 [40].

### 2.6. Determination of Reduced Glutathione

Flasks containing 2 mg of sample in 5 mL of milli-Q water were placed in an ultrasonic bath, and sonication was performed at a frequency of 50 kHz with a power of 100 W for 1 h. When finished, an aliquot of the supernatant was extracted, and the content of GSH was quantified spectrophotometrically as follows: GSH was oxidized with the sulfhydryl reagent 5,5′-dithio-bis (2-nitrobenzoic acid) (DTNB) to the yellow derivative 5′-thio- 2-nitrobenzoic acid (TNB), which was then determined at 412 nm. Then,1.2 mL of metaphosphoric acid (6%) was added to each sample (0.8 mL of the supernatant), the mixture was centrifuged at 3500 rpm for 10 min, and 250 µL of supernatant was treated with 125 µL of DTNB reagent (4 mg /mL) and 1 mL of 0.1 M of phosphate buffer, pH 7.4. After mixing thoroughly, the samples were measured at 412 nm in a spectrophotometer (Thermo spectronic, Genesys 10 UV), and the recorded absorbances were compared against a calibration curve (1–30 mg GSH/mL of sample) that allowed for calculating the content of GSH [41,42]. Sample concentration was expressed in mg of GSH per mg of complex. 

### 2.7. Statistical Analysis and Optimization Studies for the Encapsulation Process

Results were examined using analysis of variance (ANOVA), and mean separation was performed with a 95% significance level (*p* ≤ 0.05) using Statgraphics Centurium XVI. The optimization studies for the encapsulation process were performed using response surface methodology and Pareto charts.

## 3. Results and Discussion 

### 3.1. FTIR Characterization of the Alginate Complexes 

**Figure 2:**
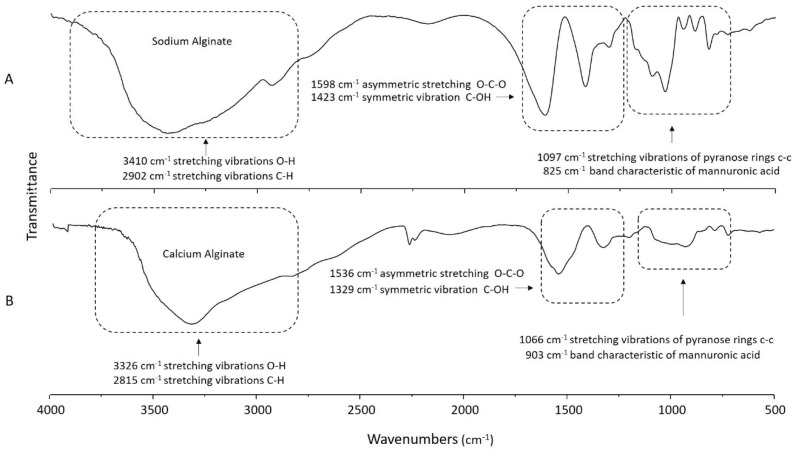
FTIR spectra of (**A**) Na-alginate, (**B**) Ca-alginate.

The FTIR analysis of Na-alginate, Ca-alginate, and the CAG complexes allowed for the examination of their functional groups and for possible interactions. The Na-alginate spectrum (Figure 2A) showed a broad band assigned to the -OH stretching vibration at 3410 cm^−1^, which changed to a narrower -OH band at 3326 cm^−1^ when Ca-alginate was analyzed (Figure 2B). The prior may be indicative of O-H stretching in the alginic acid [43] as the OH signal decreases due to the presence of calcium coordinating with the -OH groups of the alginate chains [28]. Additionally, an absorption band assigned to the -COO stretching at 1598 cm^−1^ for sodium alginate that differs from the band observed at 1536 cm^−1^ for calcium alginate was recorded (Figure 2A,B). 

The spectroscopic bands at 1536 and 1329 cm^−1^, appearing in Ca-alginate (Figure 2B), may relate to the three-dimensional network formation (“egg box” array) proposed elsewhere [44] and more specifically with the coordination of calcium, guluronic, and mannuronic acid anionic groups [45].

**Figure 3:**
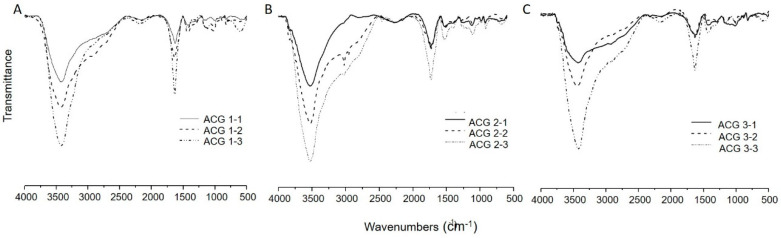
FTIR spectra of (**A**) CAG complex with 0.75% Ca and different concentrations of GSH, (**B**) CAG complex with 1.00% Ca and different concentrations of GSH, (**C**) CAG complex with 1.25% Ca and different concentrations of GSH.

As indicated before, Figure 2B and Figure 3 show a broad band assigned to the -OH stretching at 3326 cm^−1^. However, this signal overlaps with the N–H one, with a maximum at 3410 cm^−1^. In addition, the absorption bands assigned to the bending vibrations of the N–H (from GSH residues, amide band) can be observed at 1720 cm^−1^ [46], which suggested the presence of GSH within the CAG complexes (Figure 3A–C). In the GSH-containing samples, some signals of this spectrum were intensified (e.g., 3410 cm^−1^ amine group) as the concentration of GSH during sample preparation was increased (Figure 3).

### 3.2. Thermogravimetric (TG) Characterization and Differential Thermogravimetric (DTG) Analyses of CAG Complex 

**Figure 4:**
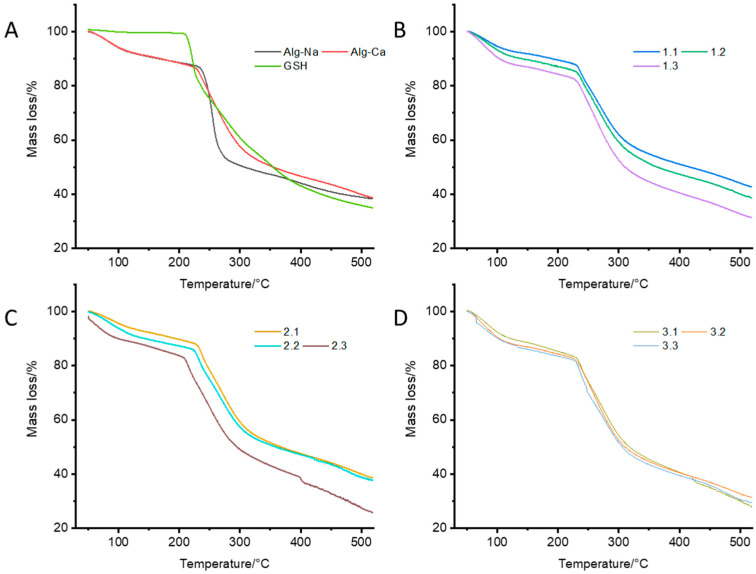
Thermal gravimetry (TG) curves of (**A**) GSH, Na, and Ca-alginate, (**B**) CAG complexes prepared with 0.75% Ca and different concentrations of GSH, (**C**) CAG complexes prepared with 1.00% Ca and different concentrations of GSH, and (**D**) CAG complexes prepared with 1.25% Ca and different concentrations of GSH.

**Figure 5:**
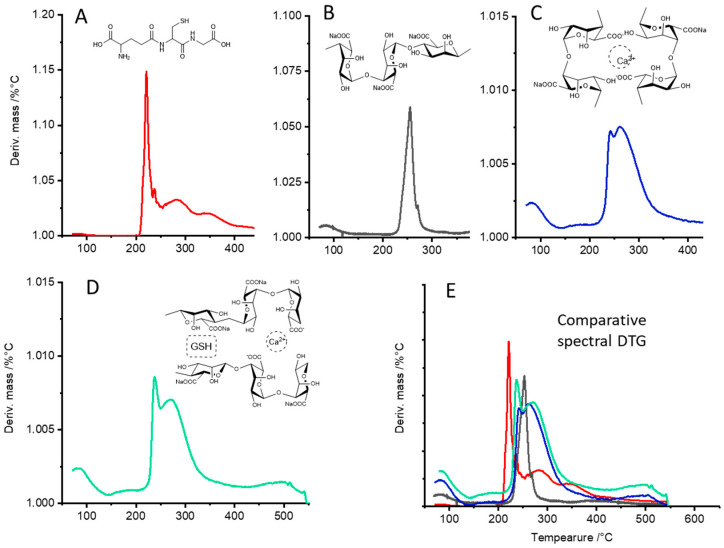
DTG curves; (**A**) glutathione, (**B**) Na-alginate, (**C**) Ca-alginate, (**D**) complex Ca-alginate-GSH, and (**E**) comparative image of overlapped curves.

TG analyses of Na-alginate, Ca-alginate, and the CAG complexes were performed to determine their thermal stability. Figure 4A shows that Ca-alginate had better thermal stability than Na-alginate, possibly due to the interactions between Ca^2+^ ions and G residues, which lead to chain−chain association and to the formation of junction zones [47]. The regions of the TG curves between 50 and 180 °C are indicative of the loss of moisture and suggest either physically weak or chemically strong bound water [48]. The results of the TG curves suggest that the hydration capacity of the complexes varied depending on their composition, and it is mainly explained by the coordination of calcium ions with water and oxygen atoms from the carboxyl chains of the alginate [49]. As seen in the DTG analyses (Figure 4B–D), the alginate fibers increased water absorption at higher calcium concentrations. Instead, when GSH was present at increasing concentrations within the complexes, a low thermal stability was observed (Figure 5), possibly due to the formation of interstitial spaces that decreased the interactions between alginate chains, thus decreasing the stability of the complexes (see Figure 1) [47,50,51] (Figure 5 B–D).

The DTG curves (Figure 5) of the different samples revealed distinctive patterns representing the first derivative of the mass loss due to thermal exposure. The CAG complex revealed two main thermal events (Figure 5D): The first region, between 50 and 180 °C, is indicative of weak and strongly bound water. Figure 5C,D shows a higher stability compared with Figure 5B, which agrees with the water absorption capacity of the fibers of Ca-alginate [28]. The second region with temperatures above 200 °C is attributed to the secondary degradation of the component of the complexes. For instance, the T_max_1 and T_max_2 of Ca-alginate appeared at 244 °C and 260 °C, respectively (Figure 5C). After mixing with GSH, the T_max_1 and T_max_2 of the samples changed to 237 °C and 268 °C (Figure 5D), possibly due to the interaction of the GSH molecules and the alginate fibers, suggesting the formation of a complex. Figure 5E shows an overlapped image of the DTG curves of glutathione, Na-alginate, Ca-alginate, and CAG.

### 3.3. Computational Building of the Molecular Structures and Polymeric Systems

Empirical evidence was used to support the experimental information regarding the gelation stability of calcium ions with the alginate fibers and the encapsulation of GSH. The analysis was based on other studies that show the capacity of the compounds to be encapsulated within the polymeric structure through non-covalent interactions [52,53].

**Figure 6:**
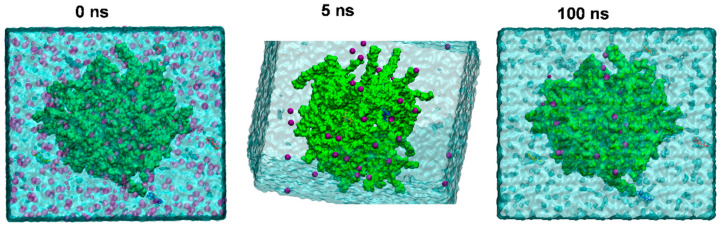
Molecular dynamic simulation of an alginate sphere structure in a water box in the presence of GSH and CaCl_2_. GSH molecules were represented in different colors, and purple dots correspond to CaCl_2_. Left to right images represent different times of the MD simulation. The center image was rotated 15° on the X axis and 20° on the Y axis in order to better observe the GSH inside the alginate sphere.

MD simulation studies were performed to determine if Ca-alginate and GSH can interact in an aqueous medium. The Na-alginate system was immersed into a water box with calcium chloride and ten GSH molecules. Figure 6 shows a general view of the evolution of the Na-alginate and calcium interaction. The simulation images show that Na-alginate adopted a more compact structure after 100 ns of simulation, with a diameter around 72 ns (Figure 6). Similar to other studies showing that the Na-alginate polymer structure can interact with Ca^2+^ molecules in an aqueous solution, in this MD simulation, Na-alginate caught the Ca^2+^ between boxes (named eggs boxes) that can be formed by GG and MM blocks. This interaction was quite fast, given that prior to the first 5 ns of simulation (Figure 6), a high number of calcium ions crosslinked with the alginate. Similarly, the image on the right shows the alginate structure after 100 ns of MD simulation where it is possible to observe a lower number of calcium ions in solution and some of the GSH molecules that did not enter the sphere.

**Figure 7:**
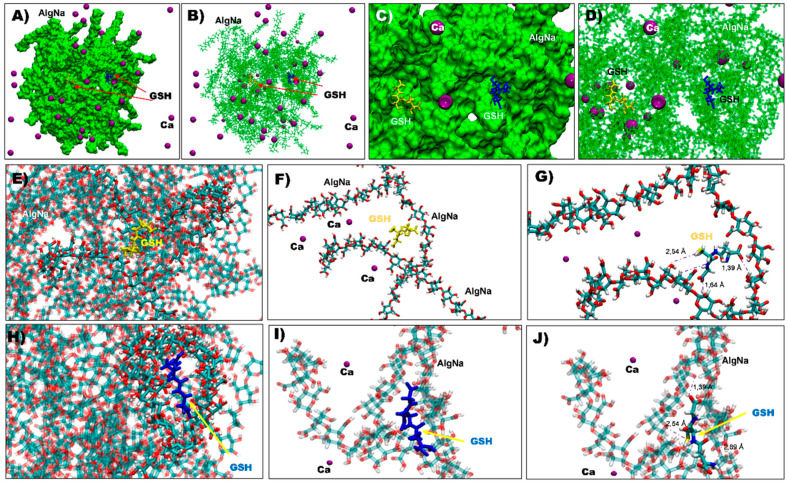
Alginate sphere structure interacting with GSH and calcium ions. (**A**) Complete view of the alginate sphere in a surface representation with two GSH molecules in the alginate, along with calcium ions interacting with the structure. (**B**) Licorice representation and the two GSH (in yellow and blue color) in the alginate sphere. (**C**,**D**) Two close views of the interaction zone of the GSH and alginates. (**E**,**H**) Two closed views of the interaction zone highlighting the monomer of alginates that interact with the GSH. (**F**,**G**) A close-up view of the first GSH that interacts with alginate. Additionally, in (**G**), the distances of the functional groups that interact between the two structures are represented. (**I**,**J**) The close-up view of the second GSH that interacts with alginate. Additionally, (**J**) shows the distances of the functional groups that interact between the two structures.

Interestingly, along with the calcium molecules that formed the complex, the sodium alginate polymer was capable to hold at least three GSH molecules in different internal empty spaces of the polymer sphere (Figure 7A–C). Thus, it can be observed how the sodium alginate structure interacts with the GSH molecules, being able to maintain a stable interaction with this molecule, showing distances around 1.5 to 3 Å (Figure 7G,J). Apparently, the GSH molecules did not interact with Ca^2+^ in the sodium alginate sphere and did so only with the monomeric alginates in the sodium alginate spaces (Figure 7F,I).

The results suggest that the GSH is encapsulated within calcium alginate, possibility by non-covalent bonding. Figure 7 shows possible physical interactions between GSH and the alginate fibers, suggesting three bonds, or interactions, as the most stable possibilities: (1) hydrogen bridge bond formation between the electrons of S in cysteine with a hydroxyl group from alginate (2.54 Å); (2) hydrogen bridge bond formation between the electrons of O in the carboxyl acid of glycine with a hydroxyl group from alginate (1.64 Å); and (3) hydrogen bridge bond formation between the electrons of N in the amine of glutamate with a hydroxyl group from alginate (1.39 Å away) (see Figure 7G,J). 

### 3.4. Determination of Reduced Glutathione

**Figure 8:**
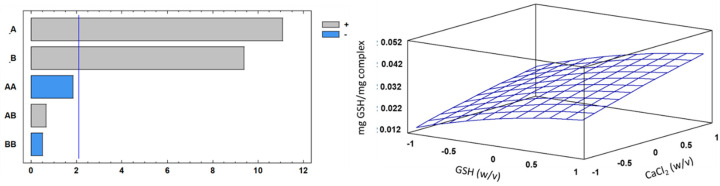
Standardized Pareto chart for mg GSH/mg complex, where A corresponds to the percentage of GSH (*w*/*v*); B, CaCl_2_ concentration (*w*/*v*); and AB, their interaction. The blue line in figure a represents the critical *t*-value, 95% confidence, while figure b corresponds to the estimated response surface for mg GSH/mg complex.

In this study, different complexes were developed by modifying the concentrations of calcium and GSH. The analysis of GSH shows that the concentrations varied from 0.014 mg of GSH (CAG 1.1) to 0.041 mg GSH (CAG 3.3). The results of Table 2 and Figure 8 show that the calcium concentration influenced the concentration of GSH within the complex at the time of gelation of the system. Thus, high concentrations of calcium allow a greater quantity of GSH molecules to be encapsulated, thereby increasing the instability of the complex due to the formation of weak bonds (intermolecular forces) between the alginate fibers and GSH [54], compared to the stability of polymers when only calcium atoms are included. In any case, the extent to which calcium or GSH concentrations can be increased outside the ranges tested in this study should be further explored. 

### 3.5. Optimization Studies for the Encapsulation Process

The goal of the optimization was to determine the conditions to maximize the glutathione content within the CAG complexes. The GSH contents obtained during encapsulation are shown in Table 2. 

The analysis of variance of the mathematical models obtained from the encapsulation results are shown in Table 3 (see Supplementary Materials). The different response functions are described in eq 1 and the response surfaces plot showing the complete regression model (R^2^ =0.91; standard error = 0.00304154) obtained for the encapsulation process. Based on the regression coefficient, the model explained 92% of the responses.

(1) $$C=\left(00301852\right)+A\left(0.00794\right)+B\left(0.00672\right)-A^{2}\left(0.002277\right)\;+\mathrm{AB}\left(0.000583\right)-B^{2}\left(0.0006111\right)$$

where: *C* is concentration (mg SGH/mg complex); A, percentage of GSH (*w*/*v*); B, CaCl_2_ (*w*/*v*). The response functions for GSH on the complex (mg) were approximated by the standard quadratic polynomial equation in Equation (1) (regression model of the system) [55].

Figure 8 shows the response surfaces and Pareto chart obtained for GSH yield after complex encapsulation. The results show that the concentrations of calcium and GSH were highly significant (*p* ≤ 0.05), indicating that higher Ca^2+^ in solution resulted in more GSH encapsulation. 

The results of the optimization protocol show that under the value coded 1 for factor A (15% *w*/*v*) and 1 for factor B (1.25% *w*/*v*), it was possible to obtain the maximum GSH concentration with a theorical result of 0.0425463 mg GSH/mg of CAG complex.

## 4. Conclusions 

A stable polymeric structure of Ca-alginate-GSH was obtained from the interaction of calcium ions crosslinking the alginate fibers (egg-box) and encapsulating GSH, possibly through non-covalent interactions between functional groups in glutathione (amine, thiol, and carboxyl acid) with hydroxyl groups from alginate. The experimental measurements were supported by molecular simulations of the structure, suggesting the interaction described. Moreover, the binding structure of metal ionic bridges between the fibers and Ca^2+^ play a major role in the stability of the complex as observed with the FT-IR and TGA-DTG analyses. 

Considering the results, an optimization model was proposed, indicating that the optimum conditions for the complex were 15% *w*/*v* GSH and 1.25% *w*/*v* CaCl_2_.

Future work should analyze the protective effect of encapsulating GHS against deleterious environmental conditions and study the release of this molecule in different matrices. 

## Figures and Tables

**Table 1:** Matrix of variables tested, coded and uncoded.

		Value		Coded	
	Samples	GSH (*w/v*)	CaCl_2_ (*w/v*)	GSH (*w/v*)	CaCl_2_ (*w/v*)
1	CAG-1.1	5	0.75	−1	−1
2	CAG-1.2	10	0.75	0	−1
3	CAG-1.3	15	0.75	1	−1
4	CAG-2.1	5	1.00	−1	0
5	CAG-2.2	10	1.00	0	0
6	CAG-2.3	15	1.00	1	0
7	CAG-3.1	5	1.25	−1	1
8	CAG-3.2	10	1.25	0	1
9	CAG-3.3	15	1.25	1	1

**Table 2:** Concentration of GSH inside the CAG complexes.

	Value		Result
Samples	GSH (*w*/*v*)	CaCl_2_ (*w*/*v*)	GSH Content (mg GSH per mg Complex)
CAG-1.1	5	0.75	0.014 ± 0.002
CAG-1.2	10	0.75	0.022 ± 0.005
CAG-1.3	15	0.75	0.027 ± 0.005
CAG-2.1	5	1.00	0.018 ± 0.002
CAG-2.2	10	1.00	0.030 ± 0.002
CAG-2.3	15	1.00	0.038 ± 0.003
CAG-3.1	5	1.25	0.026 ± 0.003
CAG-3.2	10	1.25	0.037 ± 0.001
CAG-3.3	15	1.25	0.041 ± 0.002

**Table 3:** ANOVA results for the response surface regression model on GSH encapsulation.

Source	Sum of Squares	Degrees of Freedom	Mean Square	*F*-Value	*P*-Value
A:Factor_A	0.00113606	1	0.00113606	122.80	0.0000
B:Factor_B	0.000813389	1	0.000813389	87.92	0.0000
AA	0.0000311296	1	0.0000311296	3.37	0.0823
AB	0.00000408333	1	0.00000408333	0.44	0.5144
BB	0.00000224074	1	0.00000224074	0.24	0.6282
Error	0.000175769	19	0.00000925097		
Total ss	0.00216719	26			

## Data Availability

Not applicable.

## Supplementary Materials

The following are available online at https://www.mdpi.com/article/10.3390/polym13132080/s1.
