# Supplementary material for: Design and Optimization of a Self-Assembling Complex Based on Microencapsulated Calcium Alginate and Glutathione (CAG) Using Response Surface Methodology
Source: Polymers (Basel). 2021 Jun 24;13(13):2080. doi: 10.3390/polym13132080 (PMC8271790; doi:10.3390/polym13132080)
Supplement: Supplementary file 1 [file polymers-13-02080-s001.zip › polymers-1260996-supplementary.pdf]

Design and optimization of a self-assembling complex based on microencapsulated calcium alginate and glutathione (CAG) using response surface methodology.

Table S1. Interaction effects of the treatments of. Formation of complex.

Tukey's multiple comparisons test	Mean Diff.	95% CI of diff.	Significant	Summary
1 vs. 2	-0,008000	-0.01626 to 0.0002587	No	ns
1 vs. 3	-0,0130	-0.02126 to -0.004741	Yes	*
1 vs. 4	-0,004000	-0.01226 to 0.004259	No	ns
1 vs. 5	-0,01567	-0.02393 to -0.007408	Yes	*
1 vs. 6	-0,02333	-0.03159 to -0.01507	Yes	*
1 vs. 7	-0,01167	-0.01993 to -0.003408	Yes	*
1 vs. 8	-0,02267	-0.03093 to -0.01441	Yes	*
1 vs. 9	-0,0270	-0.03526 to -0.01874	Yes	*
2 vs. 3	-0,005000	-0.01326 to 0.003259	No	ns
2 vs. 4	0,0040	-0.004259 to 0.01226	No	ns
2 vs. 5	-0,007667	-0.01593 to 0.0005920	No	ns
2 vs. 6	-0,01533	-0.02359 to -0.007075	Yes	*
2 vs. 7	-0,003667	-0.01193 to 0.004592	No	ns
2 vs. 8	-0,01467	-0.02293 to -0.006408	Yes	*
2 vs. 9	-0,0190	-0.02726 to -0.01074	Yes	*
3 vs. 4	0,0090	0.0007413 to 0.01726	Yes	*
3 vs. 5	-0,002667	-0.01093 to 0.005592	No	ns
3 vs. 6	-0,01033	-0.01859 to -0.002075	Yes	*
3 vs. 7	0,001333	-0.006925 to 0.009592	No	ns
3 vs. 8	-0,009667	-0.01793 to -0.001408	Yes	*
3 vs. 9	-0,0140	-0.02226 to -0.005741	Yes	*
4 vs. 5	-0,01167	-0.01993 to -0.003408	Yes	*
4 vs. 6	-0,01933	-0.02759 to -0.01107	Yes	*
4 vs. 7	-0,007667	-0.01593 to 0.0005920	No	ns
4 vs. 8	-0,01867	-0.02693 to -0.01041	Yes	*
4 vs. 9	-0,0230	-0.03126 to -0.01474	Yes	*
5 vs. 6	-0,007667	-0.01593 to 0.0005920	No	ns
5 vs. 7	0,004000	-0.004259 to 0.01226	No	ns
5 vs. 8	-0,007000	-0.01526 to 0.001259	No	ns
5 vs. 9	-0,01133	-0.01959 to -0.003075	Yes	*
6 vs. 7	0,01167	0.003408 to 0.01993	Yes	*
6 vs. 8	0,0006667	-0.007592 to 0.008925	No	ns
6 vs. 9	-0,003667	-0.01193 to 0.004592	No	ns
7 vs. 8	-0,0110	-0.01926 to -0.002741	Yes	*
7 vs. 9	-0,01533	-0.02359 to -0.007075	Yes	*
8 vs. 9	-0,004333	-0.01259 to 0.003925	No	ns

ANOVA test: (*) represent statistical significance at p<0.05, ns (not significance) (Tukey test).
